# Experience of using home-based fortified diet in rehabilitation of malnourished children at Indus Hospital, Karachi, Pakistan: an institution based retrospective chart review study

**DOI:** 10.1186/s40795-021-00455-x

**Published:** 2021-08-13

**Authors:** Saba Shahid, Marvi Mahesar, Anum Rahim, Yumna Sadiq

**Affiliations:** 1 Department of Pediatrics, The Indus Hospital, Karachi City, Pakistan; 2 Indus Hospital Research Center, The Indus Hospital, Karachi City, Pakistan; 3 Indus Hospital Research Center, The Indus Hospital, Karachi City, Pakistan; 4 Nutrition and Food Services Department, The Indus Hospital, Karachi City, Pakistan

**Keywords:** Malnutrition, Children, Home fortified diet, Weight gain, Anemia, And rickets

## Abstract

**Background:**

Globally, it is estimated that 50 million children under five are wasted. National nutrition survey-2018 has shown that 23.3 and 45.5% of children are wasted and stunted in Pakistan. Many studies have shown that hospital-based management of malnutrition is not practical due to high cost and iatrogenic infections and currently WHO recommends community-based management of malnutrition with provision of therapeutic food. There is limited evidence of community rehabilitation of malnourished children by using home fortified diet in Pakistan. This study explores use of energy dense, home fortified diet in achieving weight gain of malnourished children in Karachi.

**Methods:**

A descriptive, retrospective chart review of pediatric patients (aged 6 month–5 years) seen in Indus Hospital between January 2017 to June 2018 was conducted. A pre-designed data abstraction form was used to record detailed information about demographic characteristics, feeding, anthropometric, micronutrient, and nutritional details at enrollment and on follow-up.

**Results:**

A total of 361 patients were included in the final analysis. The median age (IQR) of children was 15 (14) months. Forty eight percent (*n* = 172) children had diarrhea and 54% (*n* = 195) children had respiratory tract infection. The median length of stay in the program was 28 days. The median (IQR) for average weight gain was 4.8 (0–10.3) g/Kg/day, 64.6% (*n* = 226) children defaulted, 29% (*n* = 102) were cured and 3% (*n* = 10) died.

**Conclusion:**

This study showed adequate weight gain and recovery in malnourished children by using home fortified diet in real life situations without using therapeutic food or monetary support. Home fortified diets may serve as effective strategy in community-based rehabilitation of malnourished children.

## Background

Malnutrition can be defined as a state of nutrition in which there may be excess or deficiency of calories, nutrients and proteins. Malnutrition can adversely affect normal growth, and clinical outcomes of sick children.

It was estimated that in year 2019, one hundred and forty-four million children under five were wasted globally and South Asia was the epicenter of the global burden [1]. National nutrition survey-2018 of Pakistan also reflects high burden of malnutrition in children under 5 years. Around 23.3 and 45.5% of children are wasted and stunted in Pakistan and highest rate of malnutrition is reported from Sindh province [2].

WHO initially advocated management of malnourished children in hospitals [3]. However inpatient management did not prove to be practical due to several constraints which included high cost of management, iatrogenic infections and cost to families due to prolonged hospital stay [4] Due to these challenges, WHO revised treatment guidelines and recommended that only complicated malnourished children should be treated in hospitals while uncomplicated cases should be managed in community-based settings [3]. In order to ensure optimal diet for community management, in resource-restrained countries UNICEF recommended either use of therapeutic diets like Ready to use therapeutic feed (RUTF) [5] and Corn Soya Blend (CSB) [6] or fortification of local diet with products like Micro Nutrient Powder (MNP) [7].

Products like therapeutic feed are costly. They depend on import of ingredients and presence of infrastructure for their manufacture. In Pakistan, government-run programs supply local or international produced RUTF to selected rural and urban areas [8–11]. However, the supply of RUTF is erratic and selective as a result, many urban and rural areas remain deprived of nutritional supplements. The caregivers in areas where there is no RUTF supplementation rely on unfortified indigenous food products for feeding children, which results in lack of dietary diversity and acceptable diet remains less than recommended values [2].

One logical, cost effective and feasible method of improving the quality and nutritive value of complementary food in Pakistani households is to fortify the existing recipes with locally available nutri-dense products [8]. These fortified home diets may act as surrogate for commercial therapeutic feeds and may provide a cost effective and convenient solution for nutritional rehabilitation in LMIC. We feel that feasibility, of using of fortified; home-based foods, should be checked in real life situations without giving monetary incentives to the caregivers. .

Therefore, this study aims to explore use of energy dense, home fortified diet in achieving weight gain in malnourished children through a descriptive, retrospective chart review study. The primary objective of the study was to determine cure from malnutrition on basis of weight for height standard deviation (SD) score and mid upper arm circumference (MUAC) measurement. The secondary outcomes included weight gain in g/Kg/day, average stay in nutrition program, and recovery from micronutrient deficiencies which include anemia, rickets and vitamin B12 deficiency, default rate and mortality.

## Methods

### Study area and period

Institution based descriptive, retrospective chart review was conducted from January 2017 to June 2018. The study was conducted at The Indus Hospital, which is a tertiary care facility in the suburbs of Karachi, Pakistan. Around 1200–1300 malnourished children are seen annually in this hospital, which include a mix of severe acute malnutrition (SAM) and moderate acute malnutrition (MAM). Community based rehabilitation of uncomplicated malnourished children is done in outpatient department of Indus Hospital by counseling and recommendation of fortified home-based staple diet plans with regular follow up.

### Sample size and sampling procedure

### Sample size determination

The sample size was computed using OpenEpi version 3.01 Statistical software with the following assumptions: Proportion of children attained desired weight using home based fortified diet as 69% [9], confidence level of 95% with 5% margin of error. The minimum required sample size required for the study was 329 children.

### Inclusion criteria

All children with ages ranging from 6 to 59 months with malnutrition, who were treated at Indus nutrition rehabilitation clinic (NRC), from January 2017 to June 2018 were included in the study.

### Exclusion criteria

Children who did not have proper records were excluded from the study. Children with secondary malnutrition due to other medical conditions or children who had edema due to other causes were also excluded from the study.

### Treatment protocol

Nutrition rehabilitation clinic (NRC) was conducted twice weekly in the outpatient department of Indus Hospital. The clinic catered to malnourished children with ages ranging from 6 month- 5 years. Malnutrition was diagnosed on basis of weight, height and mid upper arm circumference cut-off values prescribed by WHO. At enrollment nutritional details of each child were recorded on pilot tested, predesigned questionnaire which included nutritional history and details of physical examination. Examination was done for anthropometry and clinical features like edema, dermatosis, anemia, rickets and eye changes. If there were clinical signs of micronutrient deficiencies, then relevant labs were sent. Children with rickets and anemia were treated with oral iron and vitamin D3 supplements. The dosages of iron and vitamin D were prescribed according to WHO protocols. Blood transfusion was done in cases of severe anemia. Vitamin B12 deficiency was treated with oral Cobalamin according to institutional protocol. All the malnourished children were given multiple micronutrient powder (MNP) and zinc supplements. Antibiotics were given when needed.

Mothers were counseled on age appropriate feeding practices and hygiene strategies through Infant young child feeding practices (IYCF) counseling cards.. The diet plans were made by the nutritionist at Indus hospital using ingredients which were indigenous and available in normal households. Cooking of fortified recipes was demonstrated to the mothers in the cooking area. Quantities of the ingredients were shown by using spoon and measuring cups. After demonstration of recipes, brochures containing pictorial and written instructions in Urdu were f given to the mothers for reminder at home. Meal frequencies ranging from 2 to 6 times per day were advised based on age of child. For non-breast-fed children milk and milk products were added. About 150–220 Kcal/kg/day of calories and 3–5 g/kg/day of proteins were advised. Calories and proteins were gradually escalated in the diet.

Children were regularly followed according to severity of malnutrition. Moderately malnourished children were followed 3 weekly whereas severely malnourished children were called fortnightly. At every follow up visit mothers were asked about cooking techniques, re-counseling by the nutritionists was done if there was any variation in recipe or cooking method. Height, weight and MUAC were recorded at every follow up visit. Weight gain was calculated on basis of weight for height ratio and MUAC cutoffs. Adequate weight gain was considered when weight for height/length Z-score was equal or more than 1 standard deviation or MUAC was more than 12.5 cms. Absenteeism from follow up for more than r 6 consecutive weeks was considered as default. The children after recovery were followed for 2 months to ensure continuous weight gain.

### Operational definition

#### Severe acute malnutrition (SAM)

SAM was labeled if any of the three criteria was present (*i*) weight for height/length Z- score < − 3.0, or (*ii*) mid upper arm circumference < 11.5 cm, or (*iii*) pitting pedal edema [3].

#### Moderate acute malnutrition (MAM)

MAM was labeled when weight for height/length Z- score was <− 2.0, or (*ii*) mid upper arm circumference was between 11.5–12.5 cm [3].

#### Anemia

Severe when hemoglobin is less than 6 g/dl and moderate when hemoglobin level is between 6.1–11 g/dl [3].

#### Vitamin B12 deficiency

Plasma vitamin B 12 level < 203 pg/mL [12].

#### Rickets

Serum 25(OH) D levels at < 30 nmol/L with or without clinical signs of Rickets [13].

#### Weight gain

Weight gain was calculated in g/Kg/day. 5 g/kg/day is considered adequate weight gain [14].

#### Edema

Presence of pitting edema on dorsum of feet or shin of legs or peri-orbital edema.

#### Cure

Child was considered cured when weight for height/length SD score was > − 1.0 SD, or mid upper arm circumference was > 12.5 cm, whichever came first.

### Data collection procedure

A structured data extraction form was used for data collection. Data were gathered for demographic characteristics, feeding, and micronutrient, anthropometric and nutritional details at enrollment and on follow-up. Data were collected by nutritionists and doctor. The data extraction form was adopted from WHO guidelines [3] and Sphere standard for management of severe acute malnutrition [14].

### Data management and analysis

The statistical analysis was performed using Stata 16.0 software. Normality assessment of continuous variables was done on the basis of skewness and kurtosis. Normally distributed variables were reported as mean [SD] whereas median (IQR) was calculated for skewed variables. Paired T test was applied to compare the entry and exit variables for normally distributed variables, while Wilcoxon sign rank test was used for non-normal data. The categorical data was presented as frequencies and percentages. McNemars test was applied to measure the difference between (2 × 2) variables, whereas others with more than two categories were assed via McNemars Bowker test. *P* value < 0.05 was considered to be significant.

## Results

### Participants

**Fig. 1:**
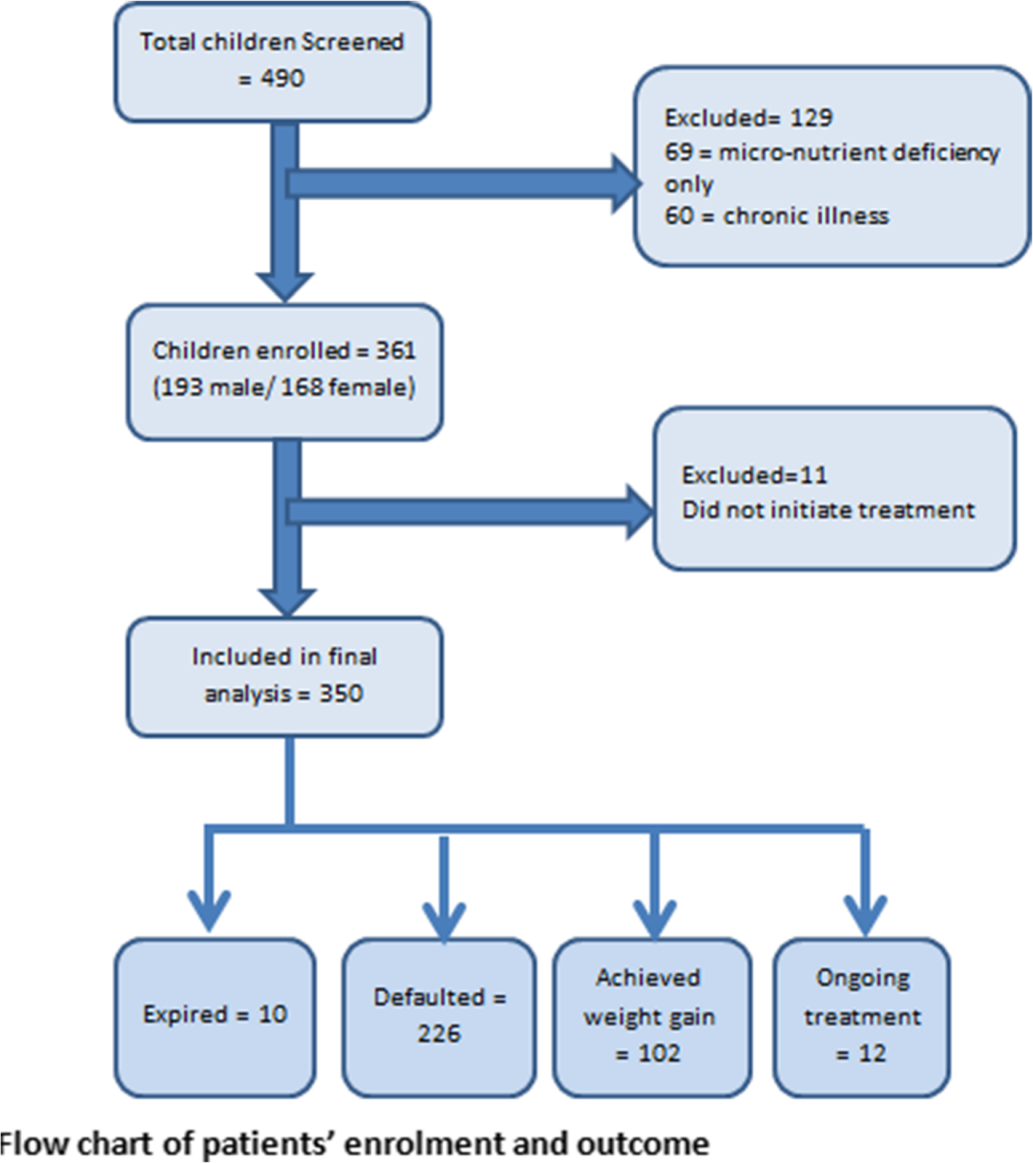
Flow-chat of patients’ enrolment and outcome

A total of 490 children were screened for malnutrition. Out of these, 129 were excluded initially due to various reasons which included presence of only micronutrient deficiencies and presence of chronic illness which could have affected clinical outcome. The data for the remaining 361 patients were further analyzed (Fig. 1).

The median age of children was 14 (IQR 15) months. More than half of children were female (53.5%, *n* = 193). Maximum numbers of children were residents of Korangi 48.9% (*n* = 175) district. Diarrhea and respiratory tract infections were most common infections, 52.8% (*n* = 161) children had diarrhea and 51.7% (*n* = 182) children had respiratory tract infection at time of enrollment. Of the 361 patients, malnutrition was identified in 97% children (*n* = 353) on basis of MUAC. Weight for height measurement identified 40% (*n* = 145) children with malnutrition (Table 1).. Eleven of the enrolled children left without initiation of treatment, thereby excluded from the final analysis (Fig. 1).

**Table 1:** Baseline characteristics of children

Variables		Frequency	Percent
Age of child* (359)	6–59	14	15
District (358)	West	7	2.0
	East	18	5.0
	Central	8	2.2
	South	9	2.5
	Malir	107	29.9
	Korangi	175	48.
	Other	34	9.5
Sex (361)	Male	168	46.5
	Female	193	53.5
Type of feeding (134)	Exclusive breast feeding	48	35.8
	Top feeding	66	49.3
	Combination feeding	20	14.9
Vitamin D deficiency (218)	Present	116	53.2
	Absent	102	46.8
Anemia (253)	Present	12	4.9
	Absent	233	95.1
Vitamin A deficiency (245)	Present	12	4.9
	Absent	233	95.1
Dermatosis (347)	Present	9	2.6
	Absent	338	97.4
Edema (349)	Present	21	6.0
	Absent	328	94.0
Otitis media (344)	Present	5	1.5
	Absent	339	98.6
Respiratory tract infection(352)	Present	182	51.7
	Absent	170	48.3
Diarrhea (305)	Present	161	52.8
	Absent	144	47.2
MUAC at enrolment(357)	Normal	4	1.1
	MAM	104	29.1
	SAM	249	69.8
Weight for height SD score at enrolment(160)	Normal	15	9.4
	MAM	51	31.9
	SAM	94	58.8

*Median (IQR)

### Analysis of primary outcomes

Out of the 350 children included in the final analysis, 64.6% (n = 226) were defaulters, 29% (n = 102) achieved adequate weight gain, 3.4% (*n* = 12) continued with the treatment and 3% (*n* = 10) died (Fig. 1 & Table 2).

**Table 2:** Outcomes at completion of study

Variables	Frequency	Percent
Duration of stay in the program* (302)	28	101
Weight gain g/Kg/day* (181)	4.8	10.3
**No of follow-ups**		
0	263	72.9
1	56	15.5
2	20	5.5
3	22	6.1
**Infection developed during treatment (98)**	39	39.8
**Cure on basis of anthropometry (102)**		
MUAC	77	75.5
Weight for height SD score	20	19.6
Both	5	4.9
**Outcome status (350)**		
defaulter	226	64.6
achieved adequate weight gain	102	29.1
expired	10	2.9
ongoing	12	3.4

*Median (IQR)

Over all 102 patients achieved adequate weight gain. Of these, 75.5% (*n* = 77) were considered to be cured on the basis of MUAC, while 20% (*n* = 20) were considered to be cured on the basis of weight for height SD score and 5% (*n* = 5) were cured on the basis of both (Table 2). There was significant difference (*p* value = 0.00) in the number of patients who were cured on the basis of MUAC and weight for height SD score (Table 3).

**Table 3:** Comparison of nutritional status at entry and exit of nutrition program

Characteristics	Nutritional status		P-value
	**At entry (%)**	**At exit (%)**	
**Nutritional status based on MUAC**			
Normal	4 (1.1)	82 (30.7)	0.00^¥^
SAM	104 (29.1)	71 (26.6)	
MAM	249 (69.8)	114 (42.7)	
**Nutritional status based on weight for height SD score**			
Normal	15 (9.4)	25 (37.3)	0.00^¥^
SAM	51 (31.9)	26 (38.8)	
MAM	94 (58.8)	16 (23.9)	
**Anemia (on basis of hemoglobin)**			
Normal	56 (15.9)	23 (21.5)	0.00^¥^
Mild Anemia	74 (21)	26 (24.3)	
Moderate	198 (56.3)	55 (51.4)	
Severe Anemia	24 (6.8)	3 (2.8)	
**Vitamin D (on basis of 25(0H) D level)**			
Normal	88 (46.6)	24 (82.8)	0.04^¶^
Deficient	101 (53.4)	5 (17.2)	
**Folate**			
Normal	54 (91.5)	11 (100)	1.00^¶^
Deficient	5 (8.5)	0 (0)	
**B 12 (on basis of serum levels)**			
Normal	104 (68)	34 (89.5)	1.00^¶^
Deficient	49 (32)	4 (10.5)	
Height*	69 (63–74.3)	72 (60–77)	0.00^€^
**Weight**	6.2 (1.7)	7.1 (1.8)	0.00^Ʈ^

*Mcnemar bowker test (*¥*),McNemars (*¶),Wilcoxon signed rank test (€), Paired T test (Ʈ), Median (IQR)*.

### Analysis of secondary outcomes

**Fig. 2:**
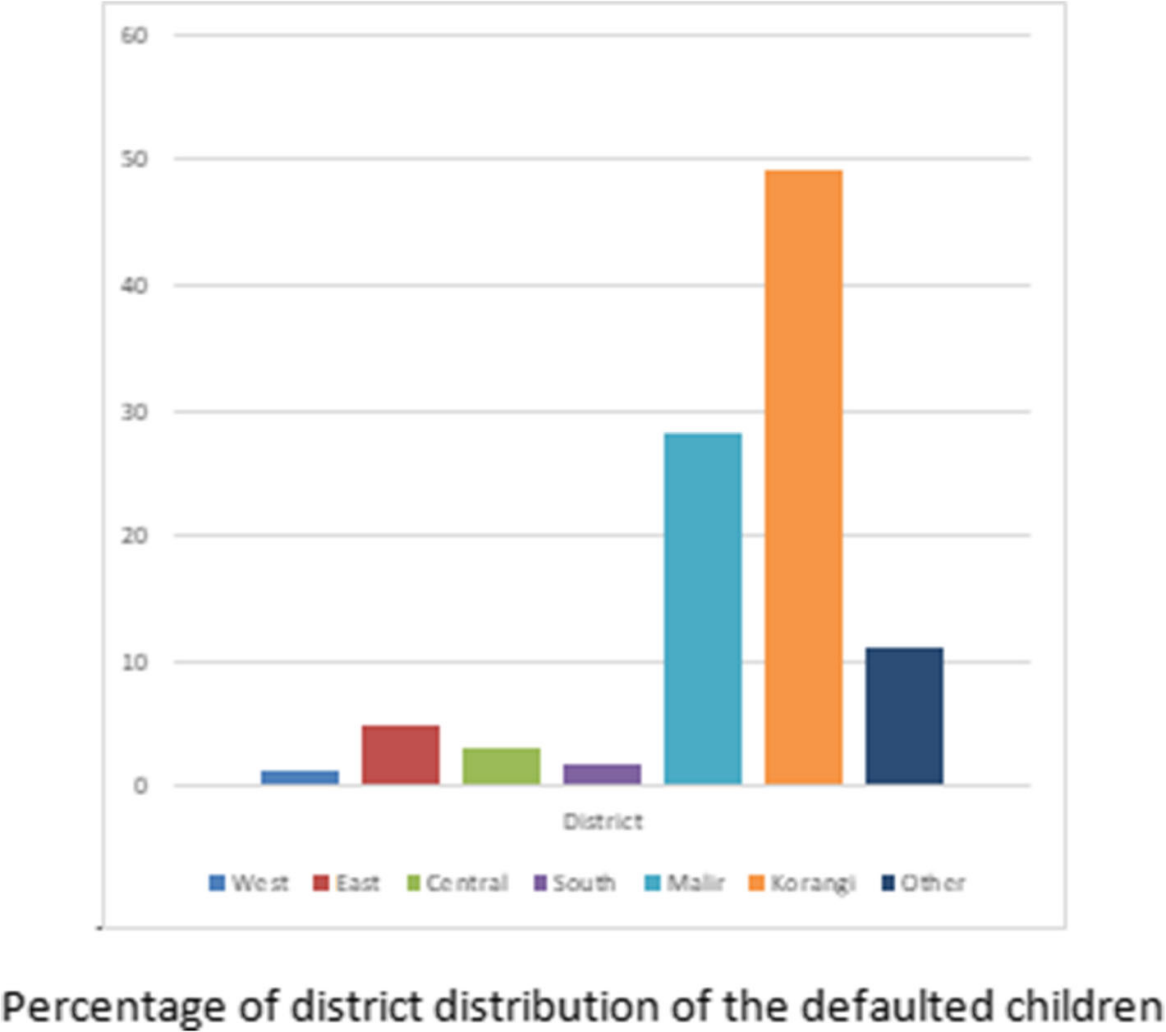
Percentage of district distribution of the defaulted children

The median (IQR) stay in the program was 28 days (101). The median weight gain was 4.8(IQR = 10.3) g/Kg/day (Table 2). Two hundred and twenty six children (65%) did not follow up in the OPDs regularly out of which 73% (*n* = 263) never came after initial visit while 22 children (6%) came for 3 visits, (Table 2). There was no significant difference between males and females in the number of defaults during follow-ups. Children who came for maximum number of follow ups belonged to Korangi district; 49% (*n* = 175). While children belonging to district West 2% (*n* = 7) had least number of follow-ups (Fig. 2).

There was significant difference in anemia and vitamin D status of the patients at the time on enrolment compared to the time of exit. Twenty-four children (6.8%) had severe anemia at time of enrollment which had reduced to 3 children (3%) at time of exit (*p*-value: 0.00). Vitamin D deficiency was observed in 101 children (53%) at enrollment while at exit 5 children (17%) had vitamin D deficiency (p-value: 0.04). Recovery from vitamin B12 and Folate deficiency were not statistically significant. Forty-nine children (32%) had vitamin B12 deficiency, out of which 45 children got cured (p-value: 1.0). Five children had Folate deficiency, and all got cured (p-value: 1.0) (Table 3).

## Discussion

This study analyzed efficacy of using fortified, energy dense, home prepared diet, in rehabilitation of uncomplicated malnourished children, aged 6 to 59 months, treated in a tertiary care hospital. Findings of this study showed that median age of children was 15 months. Out of 350 children whose final outcome was known, 65% (*n* = 226) were defaulters, 29% (*n* = 102) achieved adequate weight gain and 2.9% (n = 10) died. The average stay in nutrition rehabilitation program was 28 days. The average weight gain of the children was 4.8 g/kg/day.

In our study we were able to achieve weight gain more than 4 g/kg/day, which is WHO minimum standard for community treatment of malnourished children [15]. We did not achieve good weight gain (5–10 g/Kg/day), which is stated to be in range of 5–10 g/kg/day according to SPHERE standards [14]. The low weight gain in our study could be because of multiple factors, which may include lack of mothers’ compliance in preparation of recipes, in- frequent feeding of children, food sharing at home and unhygienic feeding practices. An additional explanation of slow weight gain in our study could be high rate of infections both at enrollment and follow up. We observed that 54 and 48% children had respiratory tract infections and diarrhea respectively. The infections could be due poor hygienic conditions and incomplete vaccination status of the children in our study.

Several studies have shown weight gain ranging from 1.7 g/kg/day to 8.7 g/kg/day with use of therapeutic, fortified diet [16–20]. However most of these studies [2–5] were done in controlled environment, where therapeutic nutrition supplements were given to the children free of cost and regular monitoring through home visits were done. We conducted this study in real life situation without any external monetary funding or food support and there were no home visits, this may have contributed to slow weight gain. We measured height and weight in every follow up and used height recorded in the last follow up for calculating final WHZ score. This was done to ensure accuracy but this method shows slow recovery from malnutrition [21] and could be a contributing factor for slow weight gain in our children.

We observed high default rate, 226 (64.6%) children did not come for regular follow ups despite frequent telephonic reminders. Aguayo and colleagues [22] also reported default rate of 11% while assessing community rehabilitation of malnutrition in Pakistan. In our study many children who defaulted, resided in close proximity to our hospital. We observed that many defaulted children continued to have infections till their last follow up. There is a possibility that parents considered infections as poor recovery and stopped coming to hospital due to this reason. Parents’ lack of understanding about adverse consequences of malnutrition in their children can be another reason for default. Baig and Mahmood have reported that parents living in urban settlements of Pakistan lack awareness about importance of nutrition due low education [23, 24]. We need further studies to determine reasons for default in hospital visits of malnourished children.

World Health Organization [15] recognizes that there is difference in group of children recognized by mid-upper-arm circumference (MUAC) and weight-for-height Z-scores (WHZ) WHO estimated that there is 40% overlap between the two indicators in identifying malnutrition [25]. We also observed difference between MUAC and WHZ scores. Seventy-seven children were cured on basis of MUAC and 20 children were cured on basis of WHZ. Grellety and Golden [26] collected data on these 2 variables from 47 countries and found out that both criteria identify different sets of malnourished children but these 2 criteria are complimentary to each other in identifying risks of mortality. Relying on one criterion may under detect cases and deny treatment to children who are at risk of dying from malnutrition related complications. The author recommended that both MUAC and WHZ should be used in nutrition programs to prevent risk of mortality and under detection of cases.

The results of our study showed that by using home fortified diet, we were able to achieve weight gain in 29% children. Weight gain of 4.8 g/Kg/day was observed. This result has important practical implication as it shows that home fortified diets if combined with robust community monitoring, can be used in place of commercially available RUTF.

### Strengths and limitations of the study

To the best of our knowledge, this is first study done in Pakistan which has assessed efficacy of home-based fortified diet in rehabilitation of malnourished children in real life situation without provision of food or financial support. This has important operational implications as many therapeutic nutrition centers in urban areas of Pakistan currently do not provide RUTF or offer home monitoring of children. We used both MUAC and WHZ for enrolment and exit which minimized risk of under detection of malnourished children.

We had some limitations due to a retrospective nature of study. These include missing records in some outcome variables. We were unable to capture important socio-demographic information like parental education and household details due to insufficient data available in files. The available information had several missing values; therefore the regression analysis was not performed.

## Conclusion

This study revealed adequate weight gain and recovery in malnourished children by using home fortified diet in real life situations without using therapeutic food or monetary support. These findings may have implications for planning future nutrition programs in Pakistan, especially in urban areas. We, therefore, recommend that robust community reach out programs, using home fortified diet and effective nutritional counseling should be implemented for rehabilitation of malnourished children.

## Data Availability

The data used in this study was retrieved from the Indus Hospital management information system, with due permission. The datasets used and/or analyzed during the current study are available from the corresponding author on reasonable request.

## Abbreviations

National nutrition survey-2018: (5)
WHO: World Health Organization
SAM: severe acute malnutrition
MAM: moderate acute malnutrition
UNICEF: United Nation Children’s Fund
RUTF: Ready to use therapeutic feed
CSB: Corn Soya Blend
MNP: Micro Nutrient Powder
IYCF: Infant young child feeding practice
